# Bortezomib potentiates antitumor activity of mitoxantrone through dampening Wnt/β-catenin signal pathway in prostate cancer cells

**DOI:** 10.1186/s12885-021-08841-1

**Published:** 2021-10-13

**Authors:** Ying Zhang, Qiuzi Liu, Wei Wei, Guoan Zhang, Siyuan Yan, Rongrong Dai, Ying Sun, Dubo Su, Shun Lv, Yong Xia, Jing Li, Changlin Li

**Affiliations:** 1 Institute of Precision Medicine, Jining Medical University, Jining, 272067 China; 2 Center for Experimental Medicine, School of Public Health, Jining Medical University, Jining, 272067 China; 3 Institute of Cancer Pathology Research, Jining Medical University, Jining, 272067 China; 4 Laboratory animal center, Jining Medical University, Jining, 272067 China; 5 Department of Histology and Embryology, School of Medicine, Nankai University, Tianjin, 300071 China

**Keywords:** Bortezomib, Mitoxantrone, Cell proliferation, Wnt/β-catenin signaling

## Abstract

**Background:**

Bortezomib (BZM), alone or in combination with other chemotherapies, has displayed strong anticancer effects in several cancers. The efficacy of the combination of BZM and mitoxantrone (MTX) in treating prostate cancer remains unknown.

**Methods:**

Anticancer effects of combination of BZM and MTX were determined by apoptosis and proliferation assay in vivo and in vitro. Expression of β-Catenin and its target genes were characterized by western blot and Real-time PCR.

**Results:**

BZM significantly enhanced MTX-induced antiproliferation in vivo and in vitro. Mice administered a combination of BZM and MTX displayed attenuated tumor growth and prolonged survival. BZM significantly attenuated MTX-induced apoptosis. Moreover, the combination of BZM and MTX contributed to inhibition of the Wnt/β-Catenin signaling pathway compared to monotherapy.

**Conclusions:**

This study demonstrates that BZM enhances MTX-induced anti-tumor effects by inhibiting the Wnt/β-Catenin signaling pathway in prostate cancer cells.

**Supplementary Information:**

The online version contains supplementary material available at 10.1186/s12885-021-08841-1.

## Background

Wnt signaling is an evolutionarily highly conserved cellular pathway that is involved in embryogenesis, development, neoplasia, cell growth, organ formation, stem cell renewal, cell cycle progression, and survival [1, 2]. Aberrant activation of Wnt/β-Catenin signaling is involved in several cancers, including colorectal cancers [3], hepatocellular carcinomas [4], melanoma [5], pancreas cancer [6], adrenocortical carcinoma [7], and prostate cancer [8]. The Wnt pathway is considered a potential therapeutic target for the development of effective tumor treatment strategies.

MTX, a type-2 DNA topoisomerase inhibitor [9], has been widely used as chemotherapy for the treatment of metastatic prostate cancer [10, 11]. Bortezomib (BZM; PS-341) is a boronic acid dipeptide that inhibits 26S proteasome activity [12], which provides clinical benefits for patients with hematological malignancies, including multiple myeloma [13] and mantle cell lymphoma [14–17]. Although BZM shows potent antitumor activity for solid tumors in preclinical studies [18], encouraging data have not been confirmed in the clinic therapies [19]. Combination treatment is often a more effective cancer treatment strategy than stand-alone treatments. Previous studies have demonstrated that the combination of BZM with other chemotherapies enhanced the clinical benefits for patients with hematological malignancies [20, 21]. However, the efficacy of the combination of BZM and MTX for prostate cancer treatment remains undetermined. In this study, we investigated whether the combination of MTX and BZM showed anti-tumor activity compared to individual treatments.

## Methods

### Cell lines, reagents, and antibodies

Human prostate cancer LNCaP, 22RV1, PC-3 cells were obtained from ATCC (Manassas, VA, USA). Cells were cultured in RPMI 1640 medium supplemented with 10% fetal bovine serum (FBS) plus 100 U/ml penicillin/streptomycin and 2 mmol/l L-glutamine. Antibodies for β-Catenin (ab32572), cyclin D1(ab16663), c-Myc (ab32072) were purchased from Abcam (Cambridge, MA, USA). β-actin (MABT825) were purchased from Sigma (Missouri, MO, USA). Alpha-tubulin (11224–1-AP), Lamin A/C (10298–1-AP), and HRP-conjugated goat anti-mouse IgG (SA00001–1) and goat anti-rabbit IgG (SA00001–2) were obtained from Proteintech Group (Chicago, IL, USA). Propidium Iodide (PI) and CCK8 kit were ordered from Beyotime (Shanghai, China).

Bortezomib (HY-10227), MG132 (HY-13259), mitoxantrone (HY-13502A) and Carboxyfluorescein diacetate succinimidyl ester (CFSE) (HY-D0938) were obtained from MCE (New Jersey, NJ). RIPA Buffer (#9806) were obtained from Cell Signaling (Danvers, MA, USA). FBS, RPMI 1640 medium, penicillin/streptomycin, and L-glutamine were obtained from Gibco (by ThermoFisher Scientific, Shanghai, China). Trizol reagent was ordered from Invitrogen (by ThermoFisher Scientific, Shanghai, China).

### Western blotting and real-time PCR

Western blotting was performed as previously described [22]. Briefly, cells were lysed using RIPA lysis buffer containing complete protease inhibitor cocktail (Roche, Switzerland). Cytoplasmic and nuclear protein were isolated using the Cytoplasmic and Nuclear Fractionation kit (Beyotime, Shanghai, China). Protein samples were subjected to SDS-polyacrylamide gel electrophoresis (SDS-PAGE) and transmembrane. The PVDF membranes were incubated with indicated primary antibodies overnight at 4 °C and then incubated with secondary antibody for 1 h at room temperature. Staining was visualized with ECL reagent (Santa Cruz Biotech).

For real-time quantitative PCR, total RNA was extracted using the Trizol reagent according to the manufacturer’s instructions. cDNAs were synthesized was performed using reverse transcription (RT) kit (Applied Biosystems, Foster City, CA). The RT products (0.5 μl) were subjected to real-time PCR using of SYBR Green. 18S rRNA was used as an endogenous control. Quantitative of SYBR Green signal was performed with LightCycler® 480 (ROCHE Diagnostic Spa, Mannheim, Germany). The relative expression level was calculated with the 2^[−∆∆Ct]^ method and expressed as a “change fold”. All data were normalized to endogenous control (*18S rRNA*) expression. The sequence of primers were designed as follows: *18 s rRNA*: sense, 5′-GAG GAT GAG GTG GAA CGT GT-3′ and antisense, 5′- GGA CCT GGC TGT ATT TTC CA-3′; *β-Catenin*: sense, 5′- GTT CAG TTG CTT GTT CGT GC-3′ and antisense, 5′- GTT GTG AAC ATC CCG AGC TAG-3′; *cyclin D1*: sense, 5′- CAT CTA CAC CGA CAA CTC CAT C-3′ and antisense, 5′-TCT GGC ATT TTG GAG AGG AAG-3′; *c-Myc*: sense, 5′-TTC GGG TAG TGG AAA ACC AG-3′ and antisense, 5′- AGT AGA AAT ACG GCT GCA CC-3′; *MMP7*: sense, 5′- TTC CAA AGT GG TCA CCT ACA G-3′; and antisense, 5′- AGT TCC CCA TAC AAC TTT CCT G-3′; *Axin2*: sense, 5′- TGT CCA GCA AAA CTC TGA GG-3′; and antisense, 5′- GTG CAA AGA CAT AGC CAG AAC-3′.

### Apoptosis analysis, cell proliferation, and cell cycle

Apoptosis was evaluated using a Dead Cell Apoptosis Kit (ThermoFisher Scientific, catalog #V13242) as previously described [23]. Briefly, 5 × 10^5^ cells treated with indicated drugs were incubated with 5 μl FITC-conjugated Annexin-V antibody and 5 μl PI for 10 min according to manufacturer’s instructions. The data was measured by flow cytometry (Beckman CytoFLEX, Germany) and analysed using the CytExpert software (Beckman Coulter, Brea, CA, USA).

Proliferation was detected by CFSE assay and CCK8 assay. CFSE is cleaved by esterase in live cells. Cleaved CFSE produces green fluorescence. The fluorescent in cells will reduce due to divide equally into daughter cells during cell division. Therefore, proliferation of cells can be tracked by fluorescent of cleaved CFSE [24]. CFSE-labelled prostate cancer cells were treated with indicated treatments for 24 h. CFSE was determined by flow cytometry. Mean fluorescence intensity (MFI) was determined by flow cytometric analysis (Beckman CytoFLEX, Germany).

CCK-8 Assay was performed as previously described. Briefly, 1 × 10^5^ cells were incubated in a 96-well culture plate. After incubation for 24 h, the cell viability was measured by a Cell Counting Kit-8 (CCK-8) (Beyotime, Shanghai, China) according to the manufacture’s protocol. The absorbance at 450 nm was determined by CytExpert software (Beckman Coulter, Brea, CA, USA).

To evaluate cell cycle, cells were stained with PI solution. Cells were fixed with cold ethanol overnight at °C followed by RNA digestion using RNase A at 37 °C for 30 min. PI fluorescence was determined by flow cytometry (Beckman CytoFLEX, Germany). Cell cycle was determined by modfitLT software (Verity Software House, Topsham, ME).

### Immunohistochemistry and scoring

The immunohistochemistry (IHC) staining procedure and scoring in our publications [22]. Briefly, tissues were fixed in 4% formalin. Paraffin-embedded tissue sections (4 μm) were subjected to dewaxing and rehydration, followed by inactivation of endogenous peroxidase activity and antigen retrieval. Tissue sections were incubated with indicated primary antibodies. Immunosignals were visualized with a DAKO LSAB System (Dako, Carpenteria, CA, USA). IHC scoring was perform as previously described [22, 25, 26].

### Proteasome activity assay

Proteasome activity was measured as previously described [27, 28]. Briefly, cells were lysed with the lysis buffer (50 mM Tris-HCl, pH 7.4, 5 mM MgCl_2_, 5 mM ATP, 1 mM DTT and 10% glycerol). Equal amount of proteins was incubated with the substrate (LLVY-AMC as chymotrypsin-like activity) for 1 h at 30 °C and the free AMC fluorescence was determined by Cytation-i5 Cell Imaging Reader (Biotek, USA).

### Animal experiments

Severe combined immunocompromised (SCID Beige) mice were acquired from Vital River Laboratory Animal Technology Co., Ltd. (Beijing, China). Mice were housed in specific pathogen-free (SPF) conditions. To construct the mice Xenograft model, prostate cancer cells were implanted subcutaneously into the flanks of 6-week-old male SCID mice. Two week after injection, mice were randomly divided into four groups and treated with vehicle, BZM (1 mg/kg, intraperitoneally, twice weekly), MTX (3 mg/kg, intraperitoneally, every day), or combination (0.5 mg/kg BZM, twice weekly; 1.5 mg/kg MTX, intraperitoneally, every day) (*n* = 14, per group). Tumor diameter was assessed every 3 days using a caliper. Testing order was randomized and blinded. Tumor growth and animal survival rate were monitored every day. Tumor volume were calculated using the following formula: [(length) x (width)^2^]/2 (*n* = 10, per group). When maximum tumor volume was close to 1500 mm^3^ were euthanized via CO_2_ inhalation. Tumors were removed from mice for IHC (i.e., Ki67 and TUNEL staining) (*n* = 4, per group). For survival cure, animal were monitored up to 65 day (*n* = 10, per group).

### Statistical analysis

Data were expressed as means ± s.e.m. Statistical significance between two groups was analyzed with unpaired Student’s t test. Differences of multiple groups were determined by one-way ANOVA analysis. Comparisons between tumor volumes were determined by two-way ANOVA analysis. Survival curves were measured with Kaplan–Meier analysis. Statistical analyses were performed with SPSS 20.0 software (Chicago, IL). *p* < 0.05 were considered significant.

## Results

### BZM enhanced MTX anti-tumor activity in vivo

**Fig. 1:**
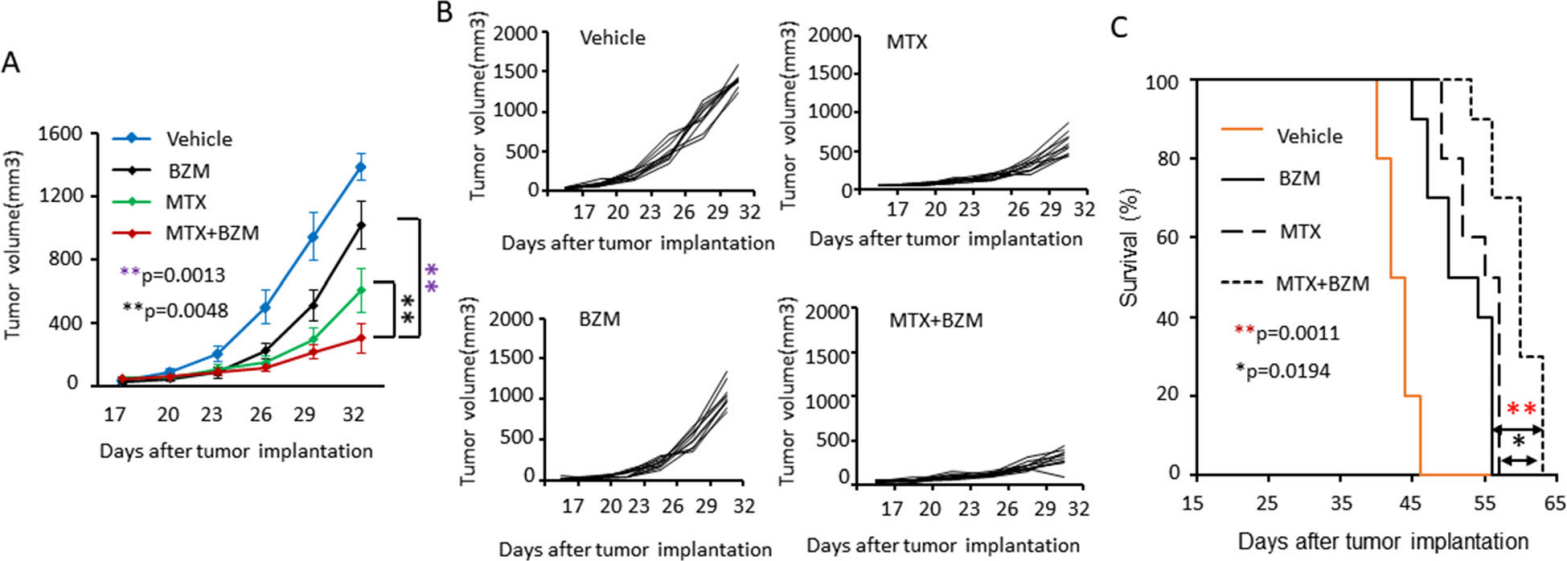
BZM enhanced MTX anti-tumor activity in vivo. **A-C**, Subcutaneous xenografts were established with LNCaP cells in male SCID mice. Two weeks after injection, mice were treated with BZM (1 mg/kg, intraperitoneally, twice weekly), MTX (3 mg/kg, intraperitoneally, every day), or combination (0.5 mg/kg BZM, twice weekly; 1.5 mg/kg MTX, every day) (*n* = 10, per group). Tumor growth and animal survival rate were monitored. **A**. Quantitative data for tumor growth curve. Tumor sizes were measured at the indicated days with a caliper and calculated as [length x width^2^]/2(*n* = 10, per group). Data are presented as means ± s.e.m. The asterisks indicate significant differences (two-way ANOVA, **p < 0.01). **B**. Tumor growth curves of individual mice (*n* = 10, per group). **C**. Overall animal survival. The asterisks indicate significant differences (*n* = 10, per group). (log-rank test, *p < 0.05, **p < 0.01)

To investigate the effect of BZM treatment on MTX-induced anti-tumor activity in vivo, we generated subcutaneous xenograft tumors with LNCaP cells in SCID mice. Fourteen days post-tumor injection, BZM, MTX, or a combination of BZM and MTX were administered by intraperitoneal injection. The combination treatment was significantly better at inhibiting tumor growth compared with individual drug treatment (Fig. 1A, B). Moreover, the combination treatment significantly prolonged survival to longer duration compared to individual drug treatment (Fig. 1C). These data strongly suggest that BZM enhances MTX anti-tumor activity in vivo.

### BZM enhanced MTX-induced anti-proliferation but attenuates MTX-induced apoptosis

**Fig. 2:**
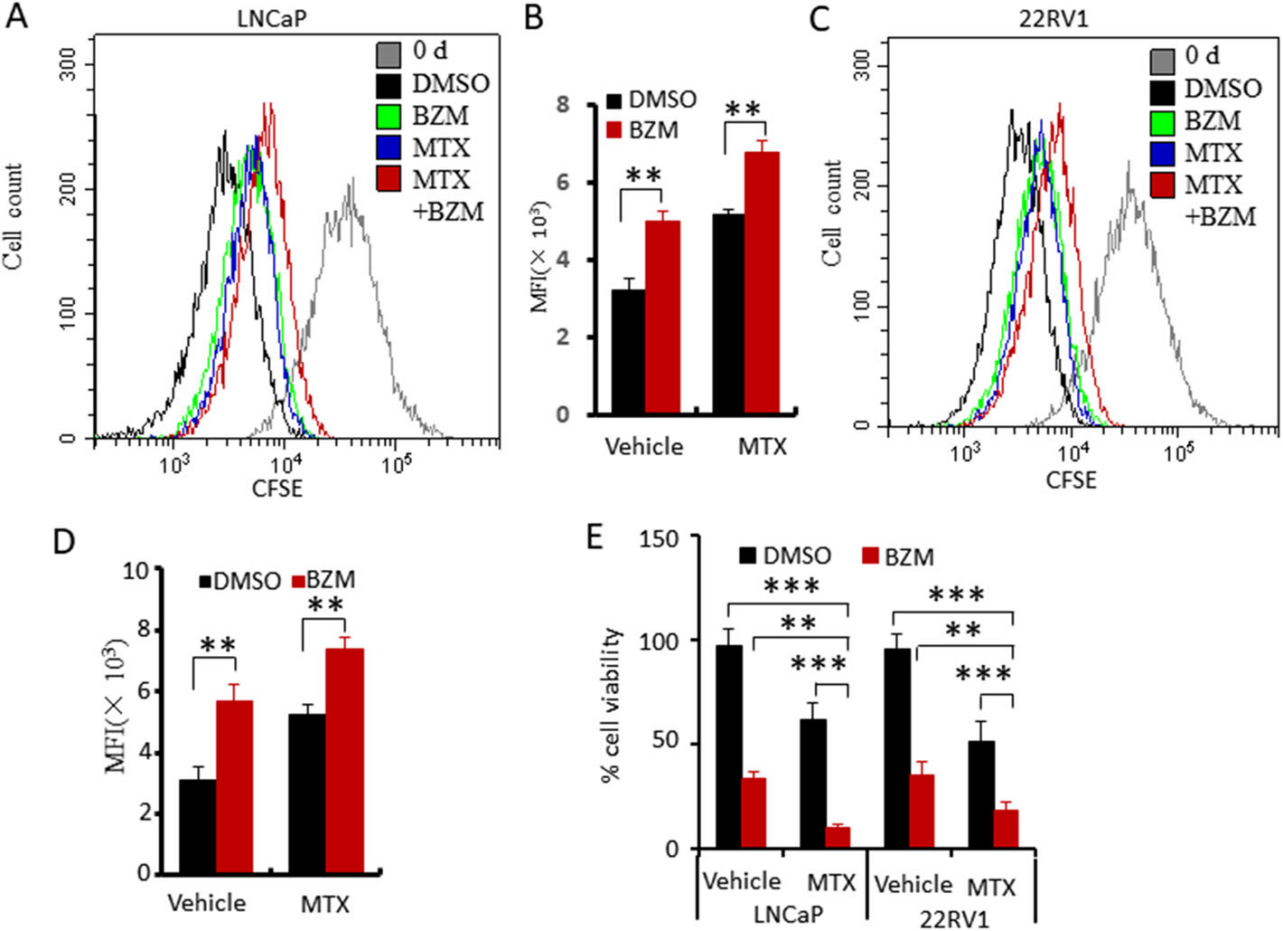
BZM enhanced MTX-induced anti-proliferation activity. **A-D**. CFSE-stained LNCaP or 22RV1 cells were treated with vehicle, MTX(1 μM), BZM (100 nM) or combination (MTX, 0.5 μM; BZM (50 nM). CFSE decay was determined by flow cytometry. **A, C**. Plots of flow cytometry. **B, D**. Quantitative data of CFSE decay. **E**. Cells proliferation was determined by CCK-8. Quantitative data were deduced from triplicate experiments and presented as means ± s.e.m. The asterisks indicate significant differences (one-way ANOVA, **p < 0.01, ***p < 0.001)

To investigate the mechanism underlying the increased anti-tumor activity of the combined BZM and MTX treatment, we examined the effect of treatment on cell proliferation using CFSE assay and CCK8 kit. LNCaP and 22RV1 cells were stained with CFSE and then treated with vehicle, BZM, MTX, or a combination of MTX and BZM. After treatment for 24 h, CFSE decay was determined by flow cytometry. The combination treatment enhanced anti-proliferation better compared to the individual treatments in both cell types (Fig. 2A, B, C, D). Similarly, CCK8 assay showed that combination treatment significantly inhibited cell viability compared with individual treatments (Fig. 2E).

We next examined the effect of treatment on cell cycle. LNCaP and 22RV1 cells were treated with vehicle, BZM, MTX, or a combination of MTX and BZM for 24 h. Cell cycles were evaluated by PI staining. The combination treatment significantly enhanced G0/G1 phase arrest in LNCaP cells (Fig. S1A, B). Similar results were observed in 22RV1 cells, and the combination treatment significantly enhanced cell cycle arrest (Fig. S1C, D).

**Fig. 3:**
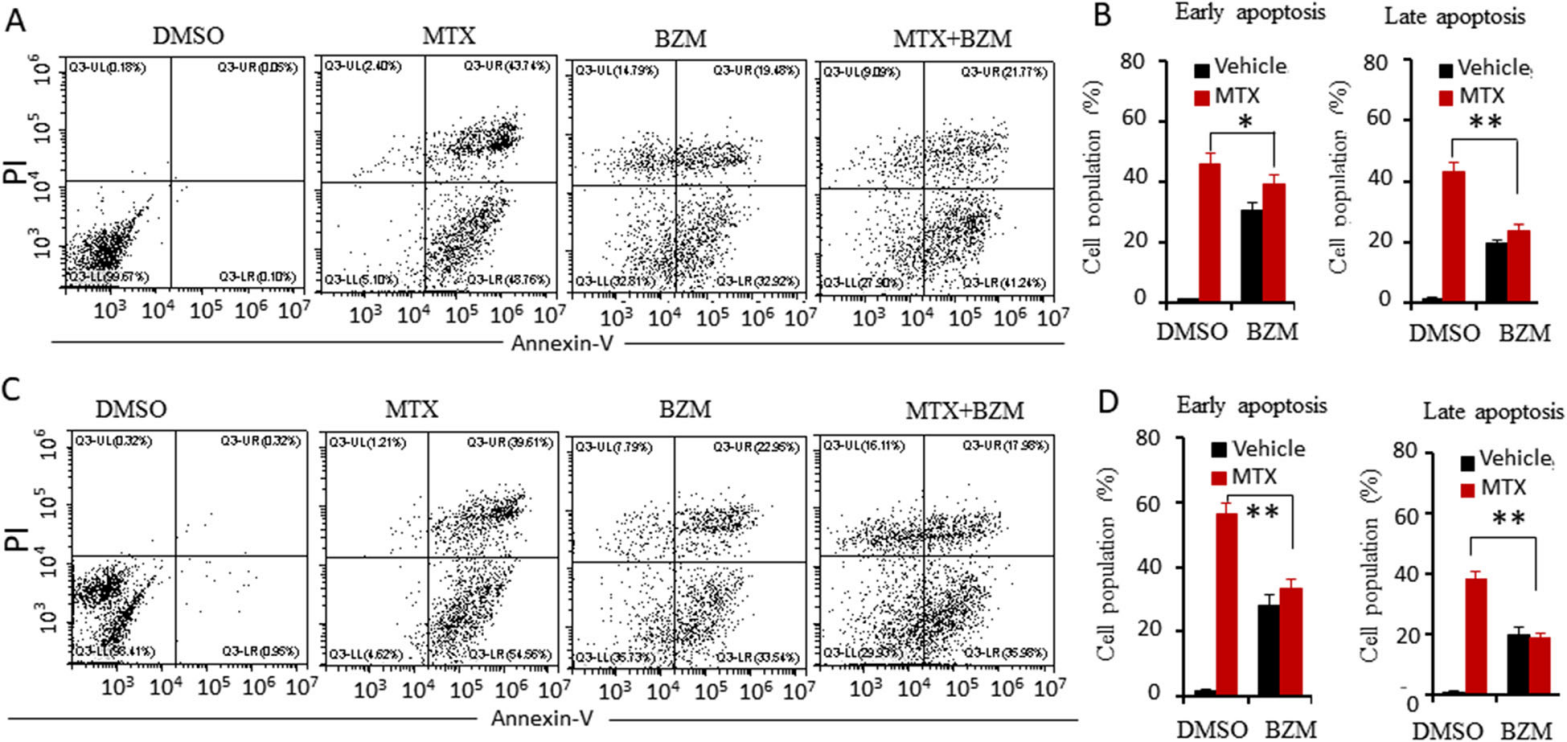
BZM attenuated MTX-induced apoptosis. **A-D**. LNCaP (**A, B**) and 22RV1(**C, D**) cells were treated with the vehicle, BZM(200 nM), MTX(1 μM) or combination(BZM, 100 nM; MTX, 0.5 μM) for 24 h. Apoptosis analysis was performed using Annexin-V/PI staining approach coupled with flow cytometry. **A, C**. Representative flow cytometry plots. **B, D**. Quantitative data of apoptosis analysis are presented from three independent experiments. Early apoptotic was defined as Annexin positive population, late apoptotic as Annexin/PI positive. The asterisk indicates a significant difference (one-way ANOVA, *p < 0.05, **p < 0.01)

To investigate whether the combination of BZM and MTX affected apoptosis, LNCaP and 22RV1 cells were treated with vehicle, BZM, MTX, or a combination of MTX and BZM for 24 h, and apoptotic cell death was evaluated using Annexin-V/PI staining coupled with flow cytometry analysis. The combination treatment significantly attenuated apoptosis to a greater extent compared to the MTX treatment in both LNCaP (Fig. 3A, B) and 22RV1 cells (Fig. 3C, D). These findings indicate that BZM enhances MTX-induced anti-proliferation, and attenuates MTX-induced apoptosis.

### BZM enhanced MTX-induced anti-proliferation but attenuated MTX-induced apoptosis in vivo

**Fig. 4:**
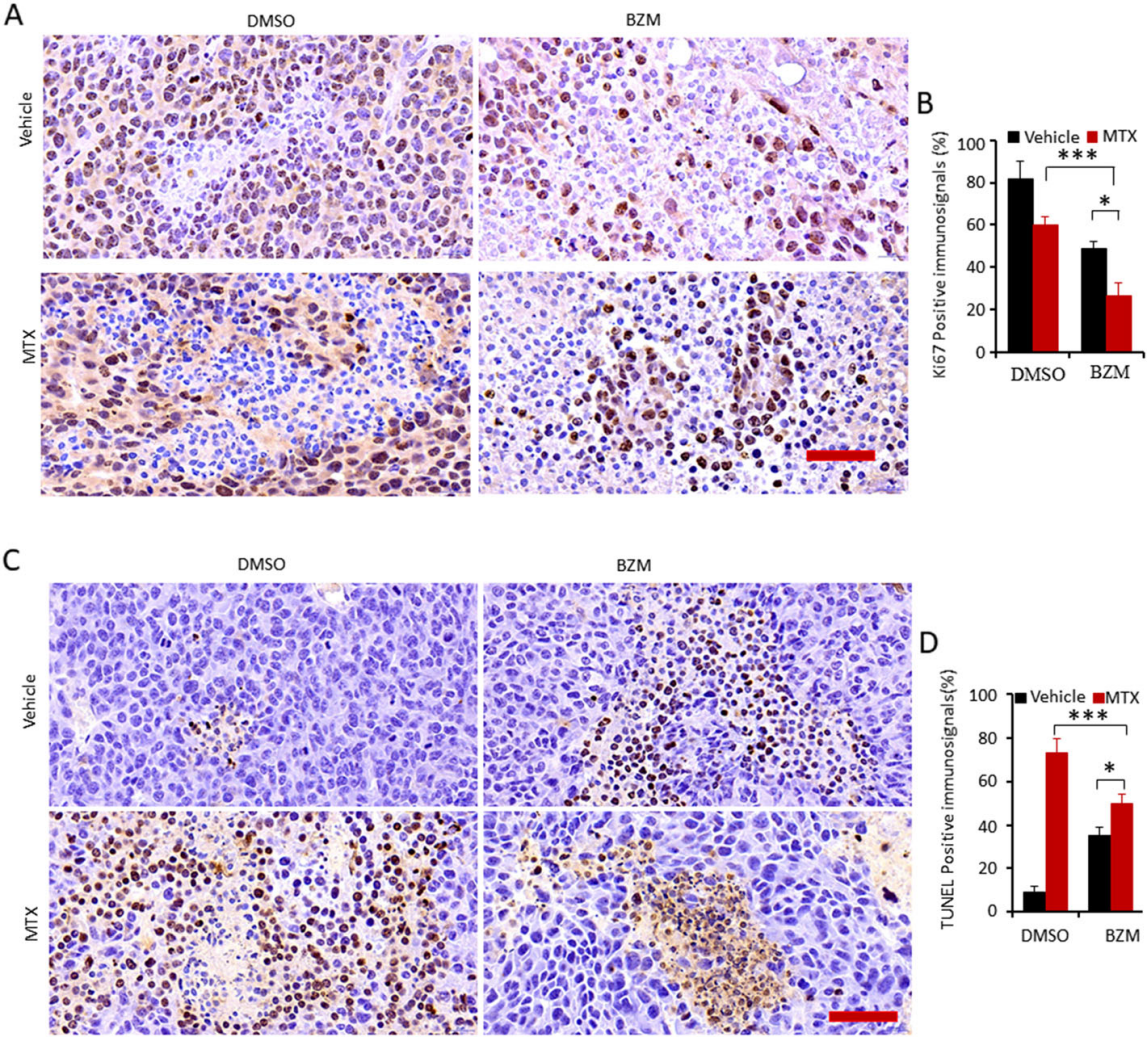
BZM attenuated MTX-induced apoptosis but enhanced anti-proliferation in vivo*.* **A-D**. Subcutaneous xenografts were established with LNCaP cells in male SCID mice and animals were treated with the vehicle (as control, n = 10), BZM (1 mg/kg), MTX (3 mg/kg) and combination of and MTX (1.5 mg/kg) and BZM (0.5 mg/kg). Mice bearing a tumor volume closing to 1500 mm^3^ were euthanized. Tumor tissues were fixed and performed IHC stain with indicated antibodies or TUNEL kit (*n* = 4, per group).**A**. Ki67 expression of tumor tissues. Representative IHC staining of Ki67, Scale bar, 50 μm. **B**. Quantitative data of Ki67 expression are presented from three independent experiments. **C**. Apoptosis were determined by TUNEL kit. Representative IHC staining of TUNEL, Scale bar 100 μm. **D**. Quantitative data of apoptosis analysis are presented from three independent experiments. All data are presented as mean ± s.e.m. The asterisk indicates a significant difference (one-way ANOVA, *p < 0.05; **p < 0.01; ***p < 0.001)

To investigate the effect of the combination treatment on the proliferation and apoptosis of prostate cells in vivo, LNCaP cells were implanted subcutaneously into the flanks of male SCID mice. Mice were administered MTX, BZM, or a combination of the two. The expression of Ki-67, a biomarker of cell proliferation, was assessed by immunohistochemical staining. The combination treatment of BZM and MTX significantly attenuated the expression of Ki-67 compared to MTX alone treatment (Fig. 4A, B). Apoptosis was assessed by the TUNEL assay. The combination treatment significantly decreased apoptosis compared to MTX alone treatment (Fig. 4C, D). These results demonstrated that the combination of MTX and BZM significantly enhanced anti-proliferation and decreased apoptosis in vivo, which is consistent with the results in vitro.

### BZM enhanced MTX-induced downregulation of nuclear β-catenin accumulation

**Fig. 5:**
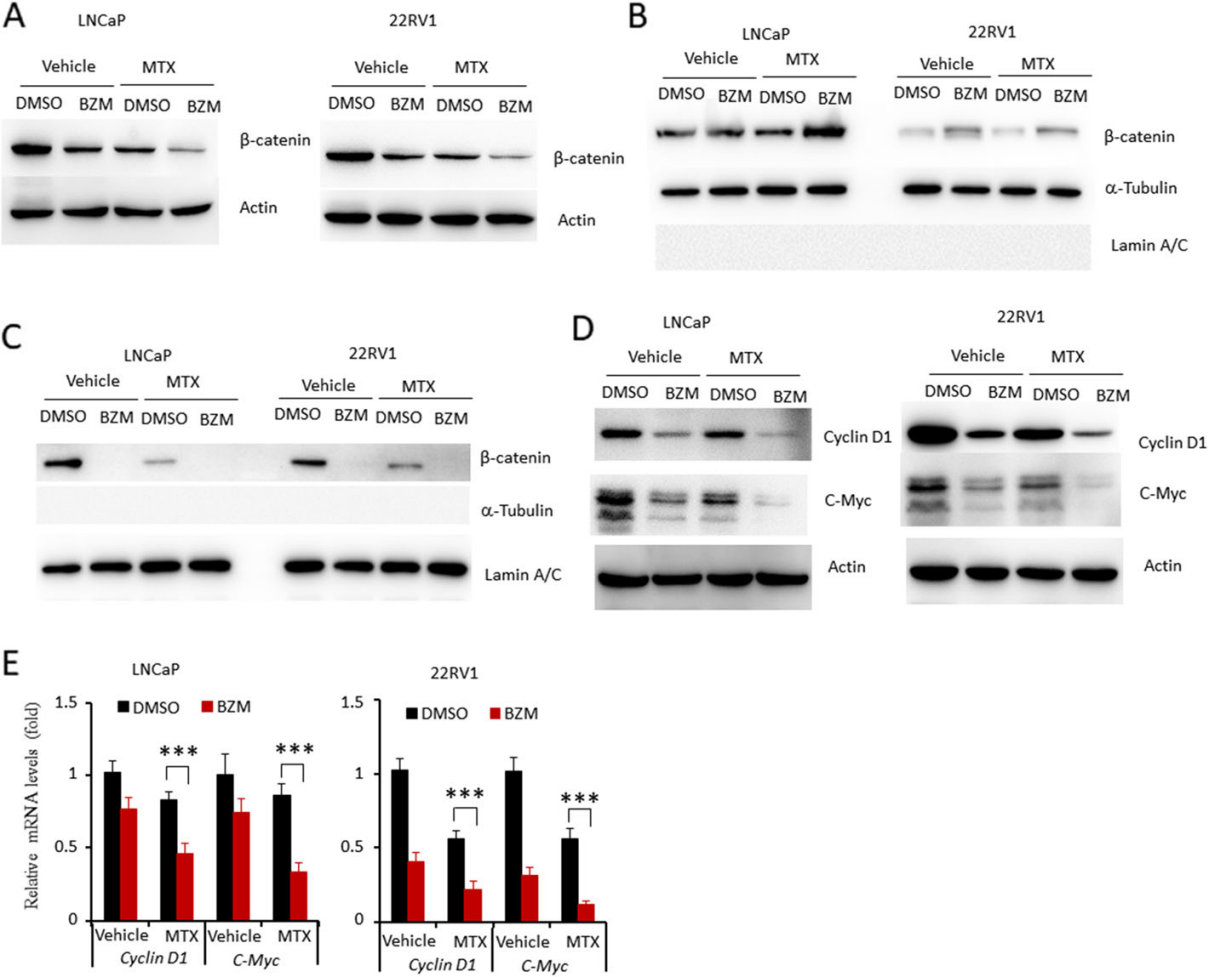
BZM aggravated MTX-induced inhibition of Wnt/β-Catenin signaling. LNCaP and 22RV1 cells were treated with vehicle, BZM (200 nM), MTX (1 μM) alone and combination (BZM, 200 nM; MTX, 1 μM) for 24 h. **A, D**. Whole cellular lysates were subjected to western blot with indicated antibodies. Actin served as endogenous protein loading control. **B, C**. Cytoplasmic (**B**) and nuclear (**C**) protein were isolated after treatment for 24 h. Expression of β-Catenin was determined by western blot. **E**. Real-time PCR assay of indicated genes expression. LNCaP(left) and 22RV1(right) cells were treated with vehicle, BZM, MTX alone or combination for 24 h. Data are presented as from three independent experiments and mean ± s.e.m. The asterisk indicates a significant difference compared to the vehicle control (one-way ANOVA, ***p < 0.001)

Although the effect of BZM on Wnt/β-Catenin pathway activity is controversial in different groups, the studies have demonstrated that Wnt/β-Catenin pathway was a critical target of BZM [29, 30]. So we investigated whether this pathway was involved in the anti-tumor activity of the combined MTX and BZM treatment. LNCaP and 22RV1 cells were treated with BZM, MTX, or a combination of MTX and BZM, and it was found that the combination treatment significantly attenuated the expression of β-Catenin protein in both cell lines (Fig. 5A). Real-time PCR analysis showed that the combination treatment did not affect the mRNA level of *β-Catenin*, in comparison to the individual drugs (Fig. S2A, B). The activity of β-Catenin is determined by the nuclear translocation of β-Catenin. We next investigated whether combined MTX and BZM treatment affect nuclear translocation of β-Catenin. LNCaP and 22RV1 cells were treated with BZM, MTX, or a combination of MTX and BZM for 24 h. Nuclear and cytosolic protein was isolated and performed for western blot. The combination treatment significantly increased cytosolic β-Catenin accumulation (Fig. 5B), while decreased β-Catenin accumulation in nucleus compared with individual drugs (Fig. 5C). Expression levels of the β-Catenin target genes were then examined. The combination treatment significantly decreased the expression of cyclin D1 and c-Myc at both the protein (Fig. 5D) and mRNA (Fig. 5E) level. Furthermore, β-Catenin target genes, *MMP7* and *Axin2* were determined by real-time PCR. Combination treatment significantly decreased expression of *MMP7* and *Axin2* compared with MTX treatment (Fig. S2C, D). Collectively, our data indicate that BZM reduces Wnt/β-Catenin signaling activity by decreasing β-Catenin protein levels in nuclear.

### Proteasome activity was required for MTX-induced apoptosis

**Fig. 6:**
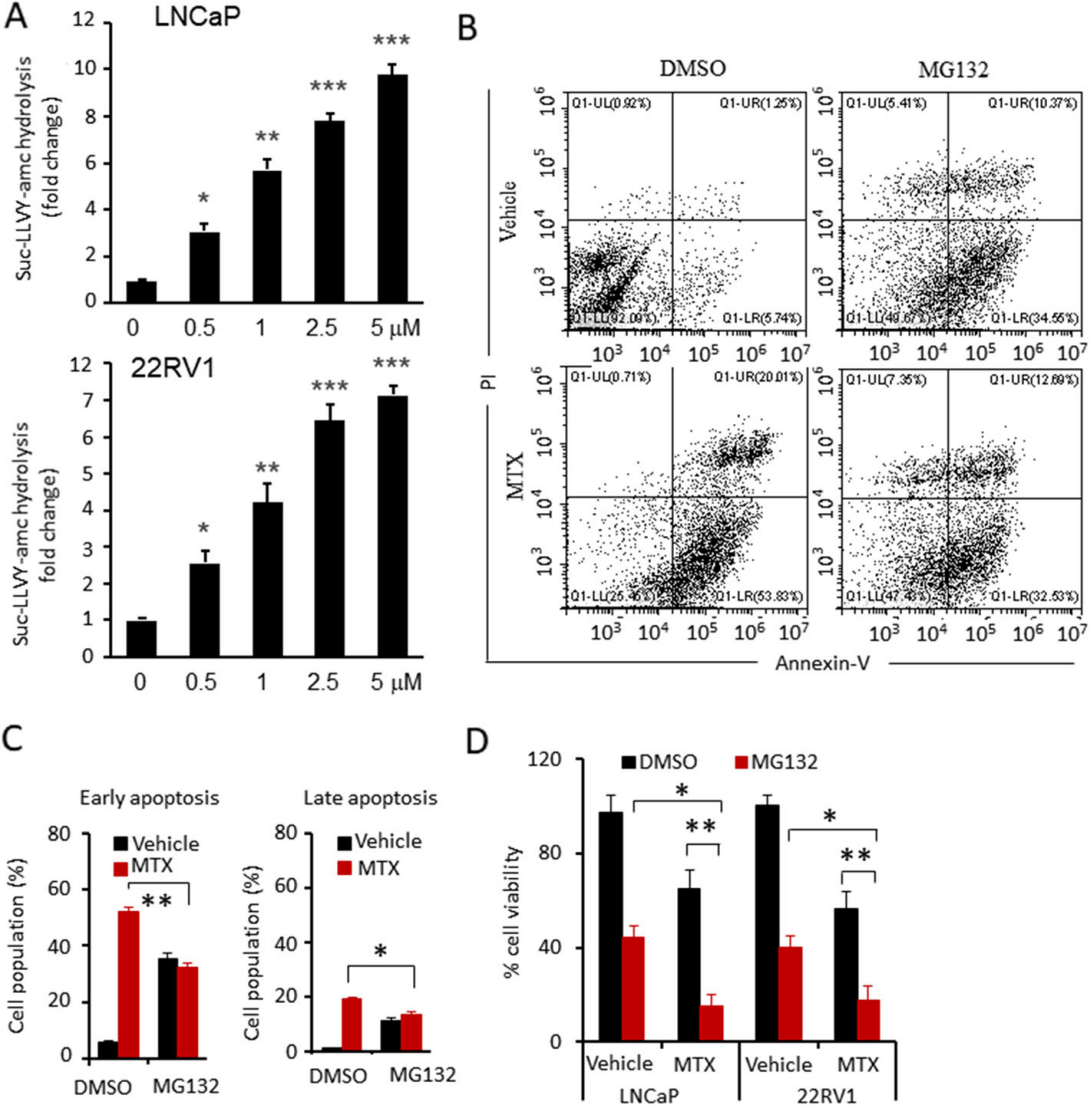
Proteasome activity was required for MTX-induced apoptosis. **A**. Proteasomes activation assay. LNCaP and 22RV1 cells were treated with vehicle, MTX (1 μM) for 24 h. Proteasome activation of LNCaP (up) and 22RV1(down) treated with indicated concentrations were indicated as hydrolysis rate succinyl-Leu-Leu-Val-Tyr-amc (Suc-LLVY-amc). **B-C**. LNCaP cells were treated with vehicle, BZM (200 nM), MTX (1 μM) alone and combination (BZM, 100 nM; MTX, 0.5 μM) for 24 h. **B**. Representative flow cytometry plots. **C**. Quantitative data of apoptosis analysis. Early apoptotic was defined as Annexin positive population, later apoptotic as Annexin/PI positive. **D**. Cells proliferation was determined by CCK-8. Data are presented from three independent experiments. All data are presented as mean ± s.e.m. The asterisk indicates a significant difference. (One-way ANOVA, *p < 0.05; **p < 0.01)

The UPS plays an important role in the cellular process of apoptosis [31], and we hypothesized that BZM attenuates MTX-induced apoptosis by interfering with the proteasome activity required for MTX-induced apoptosis. We first determined whether MTX treatment affects proteasome activity in prostate cells. MTX treatment significantly upregulated proteasome activity in LNCaP and 22RV1 cells (Fig. 6A). LNCaP cells were treated with vehicle, MTX, MG132, or a combination of MTX and MG132. Apoptotic cell death was evaluated using Annexin-V/PI staining. MG132 treatment significantly attenuated MTX-induced apoptosis either in the early or late phase (Fig. 6B, C). These data strongly suggest that proteasome activity is required for MTX-induced apoptosis, and proteasome inhibitors attenuate MTX-induced apoptosis. We also investigated whether MG132 enhanced MTX-induced anti-proliferation, LNCaP and 22RV1 cells were treated with vehicle, MTX, MG132, or a combination of MTX and MG132. MG132 treatment significantly enhanced MTX-induced anti-proliferation compared with the individual drugs (Fig. 6D).

## Discussion

Combination treatment is a good strategy to improve anti-tumor therapy. Here, we demonstrated that combination treatment with MTX and BZM is associated with greater anti-tumor effects compared to MTX or BZM monotherapy in prostate cancer. BZM significantly enhanced MTX-induced anti-proliferation both in vivo and in vitro. However, the combination of BZM and MTX attenuated MTX-induced apoptosis. Moreover, the combination of BZM and MTX significantly attenuated the Wnt/β-Catenin signaling pathway as compared to individual drug treatment.

Although BZM is currently only approved for the treatment of patients with hematological malignancies, some preclinical studies have demonstrated that BZM has strong anticancer activity in several solid tumor types [18], including prostate cancer [32]. BZM induces cell death [33, 34] and anti-proliferation [35] in prostate cancer cells. MTX is a type-2 DNA topoisomerase inhibitor that is used as a therapy for metastatic prostate cancer alone or in combination with other drugs [10, 11]. In this study, we found that BZM significantly enhanced the MTX-induced anti-tumor activity. Although BZM significantly reduced MTX-induced apoptosis, the combination of BZM and MTX inhibited tumor growth and prolonged survival both in vivo and in vitro. This may be due to the combination treatment mediating the inhibition of cell proliferation and cell cycle compared to the individual drug treatments.

Accumulating evidence indicates activation of Wnt/β-Catenin are associated with prostate tumorigenesis, metastasis, and therapy resistance [8, 36]. Wnt/β-Catenin signaling initiates prostate tumorigenesis through the induction of epithelial-mesenchymal transition (EMT) [37]. Moreover, β-Catenin interacts with androgen receptor (AR) and activates AR signaling pathway [38]. Wnt/β-Catenin signaling activation facilitates stem cell renewal [39] and contributes to resistance to therapy [40]. β-Catenin accumulated in the nucleus of enzalutamide-resistant cells and interaction of the Wnt/β-Catenin pathway overcomes resistance to enzalutamide in castration-resistant prostate cancer [41]. β-Catenin could also abolish the benefit of AR antagonist bicalutamide by increasing AR expression [42]. Inhibition of Wnt signaling can prevent prostate cancer progression [8]. While several FDA-approved drugs reportedly inhibit Wnt/β-Catenin signaling [43], inhibition of this pathway is a novel application for prostate cancer. In this study, we found that the combination of BZM and MTX significantly attenuated Wnt/β-Catenin signaling activity compared to the individual drug treatments. Some studies reported that BZM increased β-Catenin protein levels by proteasome [44]. Our result was inconsistent with the finding. Autophagy activation was able to promote the degradation of β-Catenin [45]. Both of BZM and MTX trigger autophagy in cancer cells [46, 47]. In future studies, we will investigate whether autophagy is involved in BZM-induced downregulation of β-Catenin accumulation.

Protein degradation mediated by the ubiquitin-proteasome pathway is crucial for a vast array of cellular processes, including cell death [48]. In this study, MTX increased proteasome activity in prostate cancer cells, indicating that proteasome activity may be required for MTX-induced apoptosis. BZM and MG132 attenuated MTX-induced apoptosis, which may be due to a decrease in proteasome activity.

## Conclusion

This study demonstrates that BZM enhances MTX-induced anti-tumor effects by inhibiting the Wnt/β-Catenin signaling pathway in prostate cancer cells.

## Supplementary Information

**Additional file 1 Fig. S1.** BZM attenuated MTX-induced Cell cycle arrest. **A-D** LNCaP(**A,B**) and 22RV1(**C,D**) cells were treated with vehicle, BZM (200 nM), MTX (1 μM) or combination of BZM (100 nM) and MTX (0.5 μM). Cell cycle progression was determined by PI staining. The data were deduced from triplicate experiments and presented as means ± s.e.m. The asterisks indicate significant differences (one-way ANOVA, **p < 0.01; ***p < 0.001).

**Additional file 2 Fig. S2** Expression of β-Catenin target genes. **A-D**. LNCaP (**A, C**) and 22RV1 (**B, D**) cells were treated with vehicle, BZM (200 nM), MTX (1 μM) alone or combination (BZM, 100 nM; MTX, 0.5 μM) for 24 h. Real-time PCR assay of indicated genes expression. **A, B**. Relative mRNA levels of *β-Catenin*. **C, D**. Relative mRNA levels of *MMP7* and *Axin2.* Data are presented as from three independent experiments and mean ± s.e.m. The asterisk indicates a significant difference compared to the vehicle control (one-way ANOVA, *p < 0.05, **p < 0.01).

## Data Availability

All data described in this study are available from the corresponding author upon request.
