# Supplementary figures and images for: Bortezomib potentiates antitumor activity of mitoxantrone through dampening Wnt/β-catenin signal pathway in prostate cancer cells
Source: BMC Cancer. 2021 Oct 13;21:1101. doi: 10.1186/s12885-021-08841-1 (PMC8515742; doi:10.1186/s12885-021-08841-1)

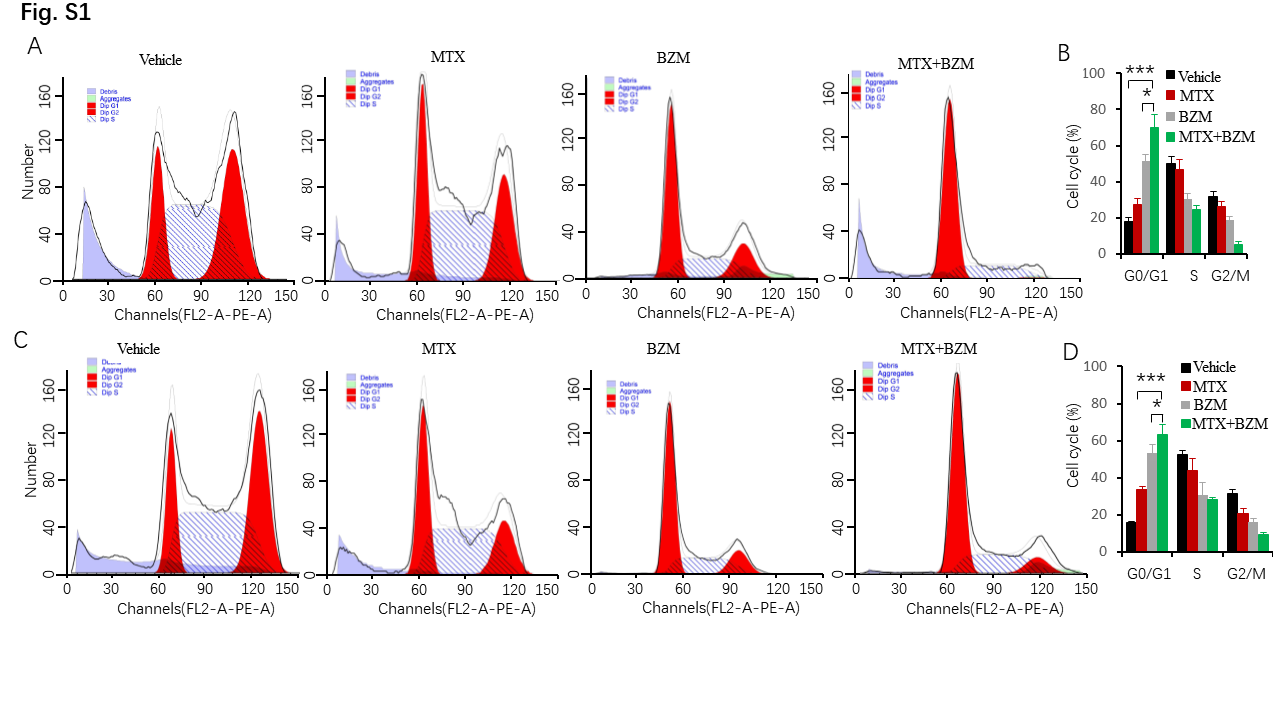

Supplement: Supplementary file 1 — Additional file 1 Fig. S1. BZM attenuated MTX-induced Cell cycle arrest. A-D LNCaP(A,B) and 22RV1(C,D) cells were treated with vehicle, BZM (200 nM), MTX (1 μM) or combination of BZM (100 nM) and MTX (0.5 μM). Cell cycle progression was determined by PI staining. The data were deduced from triplicate experiments and presented as means ± s.e.m. The asterisks indicate significant differences (one-way ANOVA, **p < 0.01; ***p < 0.001). [file 12885_2021_8841_MOESM1_ESM.tif]

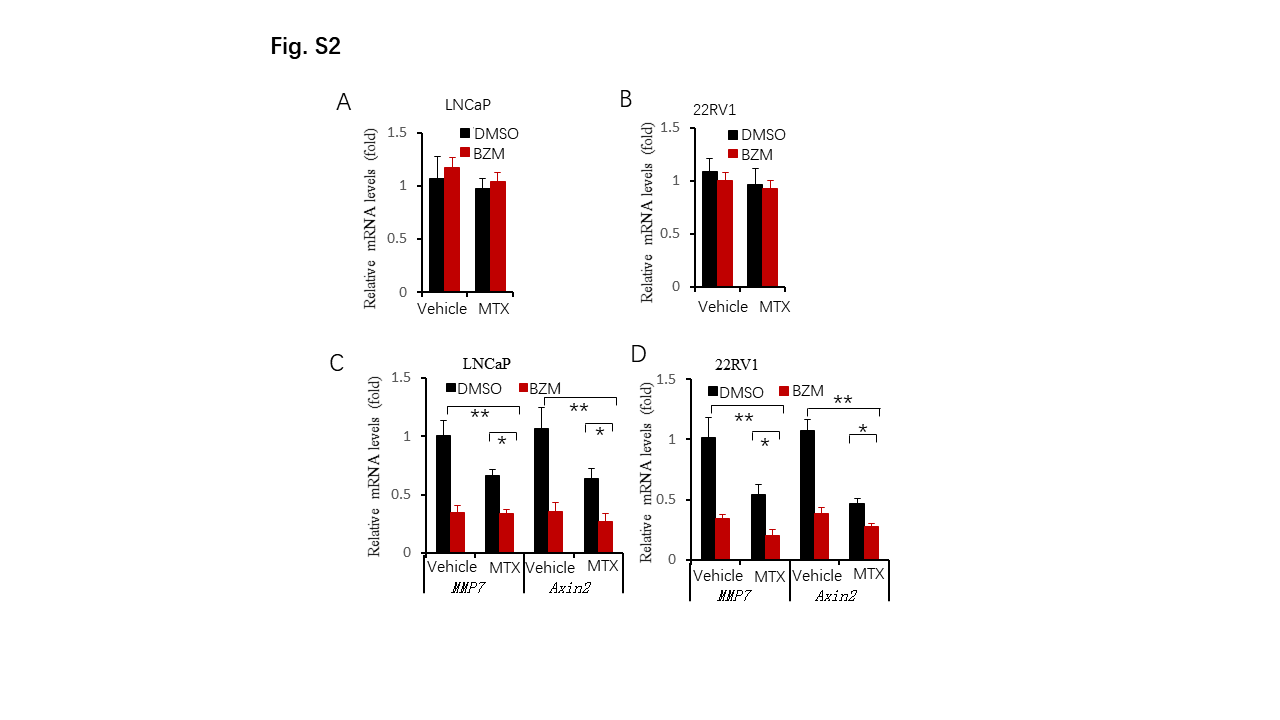

Supplement: Supplementary file 2 — Additional file 2 Fig. S2 Expression of β-Catenin target genes. A-D. LNCaP (A, C) and 22RV1 (B, D) cells were treated with vehicle, BZM (200 nM), MTX (1 μM) alone or combination (BZM, 100 nM; MTX, 0.5 μM) for 24 h. Real-time PCR assay of indicated genes expression. A, B. Relative mRNA levels of β-Catenin. C, D. Relative mRNA levels of MMP7 and Axin2. Data are presented as from three independent experiments and mean ± s.e.m. The asterisk indicates a significant difference compared to the vehicle control (one-way ANOVA, *p < 0.05, **p < 0.01). [file 12885_2021_8841_MOESM2_ESM.tif]
